# Enhanced Eating Competence Is Associated with Improved Diet Quality and Cardiometabolic Profile in Finnish Adults with Increased Risk of Type 2 Diabetes

**DOI:** 10.3390/nu13114030

**Published:** 2021-11-11

**Authors:** Kirsikka Aittola, Leila Karhunen, Reija Männikkö, Elina Järvelä-Reijonen, Santtu Mikkonen, Pilvikki Absetz, Marjukka Kolehmainen, Ursula Schwab, Marja Harjumaa, Jaana Lindström, Timo Lakka, Tanja Tilles-Tirkkonen, Jussi Pihlajamäki

**Affiliations:** 1 Institute of Public Health and Clinical Nutrition, School of Medicine, University of Eastern Finland, 70211 Kuopio, Finland; Leila.Karhunen@uef.fi (L.K.); Reija.Mannikko@terveystalo.com (R.M.); Elina.Jarvela-Reijonen@uef.fi (E.J.-R.); Pilvikki.Absetz@gmail.com (P.A.); Marjukka.Kolehmainen@uef.fi (M.K.); Ursula.Schwab@uef.fi (U.S.); Tanja.Tilles-Tirkkonen@uef.fi (T.T.-T.); Jussi.Pihlajamaki@uef.fi (J.P.); 2 Endocrinology and Clinical Nutrition, Department of Medicine, Kuopio University Hospital, 70029 Kuopio, Finland; 3 Department of Applied Physics, University of Eastern Finland, 70211 Kuopio, Finland; Santtu.Mikkonen@uef.fi; 4 Collaborative Care Systems Finland, 00270 Helsinki, Finland; 5 VTT Technical Research Centre of Finland Ltd., 02044 Espoo, Finland; Marja.Harjumaa@salivirta.fi; 6 Department of Public Health and Welfare, National Institute for Health and Welfare, 00271 Helsinki, Finland; Jaana.Lindstrom@thl.fi; 7 Institute of Biomedicine, School of Medicine, University of Eastern Finland, 70211 Kuopio, Finland; Timo.Lakka@uef.fi; 8 Department of Clinical Physiology and Nuclear Medicine, Kuopio University Hospital, 70029 Kuopio, Finland; 9 Kuopio Research Institute of Exercise Medicine, 70100 Kuopio, Finland

**Keywords:** adiposity, diet, diet quality, eating behavior, glucose metabolism, health care, lifestyle, lipid metabolism, obesity, overweight, prevention, type 2 diabetes

## Abstract

Eating competence (EC) is characterized by positive attitudes towards food and eating, having regular meals, eating a variety of foods, and internally regulated eating. We investigated the associations of changes in EC with changes in lifestyle, anthropometrics and biomarkers of glucose and lipid metabolism in 2291 adults at increased risk of type 2 diabetes as part of the StopDia study conducted in primary healthcare. EC and diet quality were assessed with validated digital questionnaires. During the intervention, the participants received either (1) the digital lifestyle intervention, (2) the combined digital and face-to-face group-based lifestyle intervention, or (3) standard care. EC increased among the participants independent of the intervention type. Increase in EC was associated with an increase in diet quality, high-density lipoprotein (HDL) cholesterol, and with a decrease in body mass index and waist circumference, regardless of baseline EC. Of the subdomains of EC, the contextual skills, food acceptance and eating attitudes were associated with various of these changes. Our results thus suggest that EC could be a potential target in lifestyle interventions aiming to improve the cardiometabolic health of people at type 2 diabetes risk.

## 1. Introduction

Type 2 diabetes (T2D), a major health and economic burden in the world, can be prevented among those at increased risk with lifestyle interventions that include intensive nutrition and physical activity counselling [1,2,3,4]. However, the impact of these interventions depends on how well new lifestyle habits, including a health promoting diet, are adopted [5]. Moreover, the role of changes in eating behavior remains unclear. A novel approach to support healthy lifestyle could be to focus on eating competence (EC) as defined in the Satter Eating Competence Model [6,7,8,9]. According to ecSatter, EC comprises an individual’s flexible and positive attitudes towards food and eating, acceptance of a variety of foods, internal regulation of eating and contextual skills around daily meals [6].

The EC has been linked to many health-related parameters, such as an improved diet quality [7,8,10,11,12,13] and weight management in different populations [7,10,13,14,15]. Our previous study showed that being eating competent was strongly associated with a better diet quality, a decreased likelihood of being obese or having an increased waist circumference, metabolic syndrome or newly diagnosed T2D in adults at increased risk of T2D [9]. However, so far, previous findings have mainly been supported by cross-sectional studies.

Eating attitudes and behaviors begin to develop during childhood and change throughout life [6] suggesting that EC can potentially be influenced by lifestyle interventions. However, to the best of our knowledge, there have so far been only a few studies dealing with the effects of lifestyle interventions on EC. One of these studies showed that EC increased and was associated with reduced body weight during a 1-year lifestyle intervention aimed at weight loss [12]. However, no changes in EC were found in other two 8-week interventions [16,17], but after a 32-week follow-up one subdomain of EC, food acceptance, improved in the intervention consisting of group-based acceptance and commitment therapy [17]. On the other hand, it is noteworthy that some aspects of EC, such as eating attitudes and internal regulation, might even decrease temporarily during lifestyle intervention [12], most likely due to increased awareness of those features. Thus, the results of earlier studies suggest that EC could be influenced by lifestyle interventions. Nevertheless, there is no previous evidence on the associations of changes in EC with changes in cardiometabolic risk factors.

Therefore, we utilized the data from the Stop Diabetes (StopDia) study [18] to investigate whether EC changed during a 1-year lifestyle intervention in adults at increased risk of T2D. We then examined whether changes in EC were associated with changes in diet quality, physical activity, overall adiposity, abdominal adiposity or glucose and lipid metabolism.

## 2. Materials and Methods

### 2.1. Study Design

This study is a secondary analysis of the StopDia study that included a 1-year parallel randomized controlled trial (RCT) (ClinicalTrials.gov registration no. NCT03156478) investigating the effects of a lifestyle intervention delivered via digital app and group counselling on risk factors for T2D in individuals at risk of T2D [18]. A detailed protocol of the StopDia study has been described earlier [18]. The StopDia study was conducted in primary healthcare as part of its routine actions between April 2017 and February 2019. The participants with entire data on EC were included in the study.

### 2.2. Participants

The participants at increased risk of T2D were recruited to the StopDia study using the StopDia Digital Screening Tool [18], including questions from the Finnish Diabetes Risk Score (FINDRISC) [19]. The inclusion criteria for the StopDia study were: (1) age of 18 to 70 years; (2) at least 12 points in the FINDRISC or previous gestational diabetes or repeated impaired fasting glucose or impaired glucose; (3) residence in one of the study provinces (Northern Savo, Southern Carelia or Päijät-Häme); (4) possibility to use a computer or smart device with an Internet connection; (5) having a personal cell-phone number; and (6) adequate Finnish language skills. Individuals with previously diagnosed type 1 or 2 diabetes, pregnancy or current cancer treatment were excluded [18].

**Figure A1:**
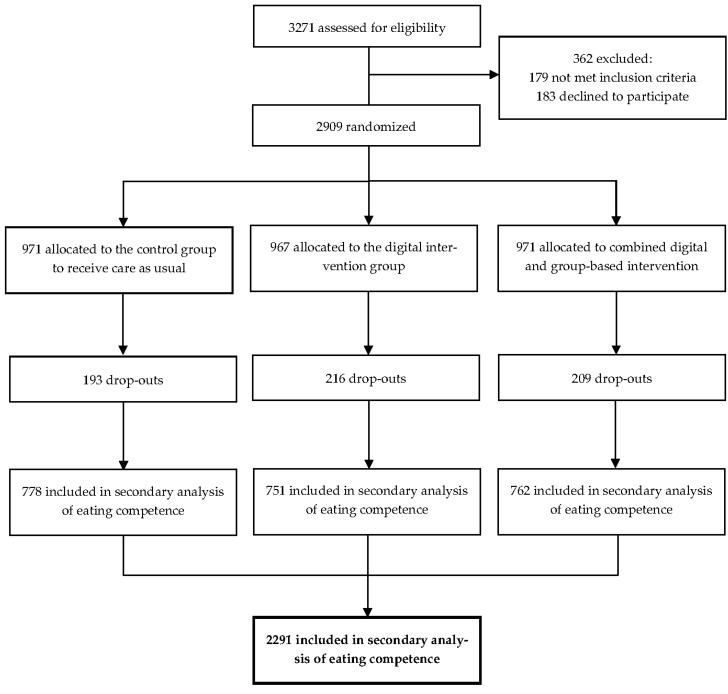
Flow chart describing how the study population of the present study (altogether 2291 participants, see the last row of the figure) was formed from the whole StopDia population (*n* = 2909 [18]).

First, potential participants according to the screening tool were invited to participate in the study and were referred to a nurse’s visit at their local primary healthcare center [18]. There a nurse checked that the participants were suitable for the study, the participants signed an informed consent and a trained nurse performed clinical measurements and referred the participants to laboratory measurements at local laboratories. Additionally, the participants completed online the StopDia Digital Questionnaire (LimeSurvey GmbH, Hamburg, Germany). Finally, 2909 eligible individuals were digitally randomized to the StopDia study: 967 of them were randomized to the digital, 971 to the combined digital and group-based lifestyle intervention, and 971 to the control groups (Figure A1). After randomization, the participants were informed by e-email of their study group as well as the content of the intervention [18].

Participants with missing data on EC either at baseline or at 1 year were excluded from the analyses (*n* = 618, 21%). Therefore, altogether 2291 participants comprised the population of this study (Figure A1). Those who were excluded were younger (51 vs. 56 years), less educated (60% vs. 67% with highest education), and less eating competent (29% vs. 40%) and had a higher BMI (32 vs. 31 kg/m^2^) at baseline compared to those who participated to assessments at both time points (all *p* < 0.05).

### 2.3. Lifestyle Interventions

The general lifestyle goals of the intervention were to improve diet, increase physical activity, decrease sedentary behavior, improve sleep, cease smoking, and moderate alcohol consumption [18].

Both intervention groups (digital; combined digital and group-based) obtained access to the BitHabit app and were instructed to use it throughout the one-year intervention period [18,20]. The habit formation and self-determination theories-based app included nearly 500 simple behavioral suggestions intended to support habit formation for a healthier lifestyle, including eating behaviors and attitudes. The participants were able to browse, select, and track habits from different lifestyle categories.

The combined digital and group-based group additionally received lifestyle group counselling that included six pre-structured group meetings to support behavior change and was based on the self-determination theories. The group meetings were organized in local healthcare centers by trained nurses or nutritionists. Between the meetings, lifestyle changes were supported by homework materials. Some aspects included in the ecSatter were considered in the digital and in the group-based intervention such as paying attention to eating and meal rhythm, planning meals, eating a variety of foods, experimenting new foods, and having flexible attitude towards food. In addition, group meetings addressed themes such as daily rhythm, health-promoting diet, exercise, lifestyle changes, and success in them [18].

The control group received brief written information about lifestyle risk factors of type 2 diabetes and recommendations on a healthy diet and physical activity. Participants in all groups obtained the results of their own laboratory measurements after their laboratory visits at baseline and 1-year follow-up.

At the end of the intervention, participants were reminded to book an appointment for the 1-year follow-up visits, which included the same measurements as at the baseline.

### 2.4. Assessments

#### 2.4.1. Eating Competence (EC)

EC was measured with the 16-item Satter Eating Competence Inventory 2.0^TM^ (ecSI 2.0^TM^) [6,7,10,21,22], which has been validated among general and low-income US people [7,22,23] and used in intervention studies among people with obesity [12,16], also in Finland [17,24]. The inventory contains six items to determine eating attitudes (e.g., ’I enjoy food and eating’), five to contextual skills around meals (e.g., ’I have regular meals’), three to food acceptance (e.g., ‘I experiment with new food and learn to like it’), and two to internal regulation of hunger and satiety (e.g., ’ I eat until I feel satisfied’). The response options for the items were always, often, sometimes, rarely and never, and scored 3, 2, 1, 0 and 0, respectively [22]. Items were summed to yield a total score of EC ranging from 0 to 48 and to yield four sub-scores [25]. A person scoring 32 or more was defined as an eating competent one [22]. Cronbach’s alpha coefficient was 0.84 for the total score of ecSI 2.0^TM^, 0.77 for eating attitudes, 0.76 for contextual skills, 0.62 for food acceptance and 0.72 for internal regulation at baseline. The respective values at 1-year follow-up were 0.85, 0.78, 0.77, 0.65 and 0.76.

#### 2.4.2. Diet Quality

Quality of diet was assessed by the Healthy Diet Index (HDI) [26]. The HDI assesses the adherence to a healthy diet according to the Finnish nutrition recommendations [27] and prevention of T2D with a scale ranging from 0 (lowest quality) to 100 (highest quality). It comprises of the following seven domains, which are weighted depending on their importance in a diet to prevent T2D: 1. meal pattern (score range 0–10); 2. fruit and vegetables (0–20); 3. grains (0–20); 4. fats (0–15); 5. fish and meat (0–10); 6. dairy (0–10); and 7. snacks and treats including beverages (0–15) [26].

#### 2.4.3. Physical Activity

The total physical activity was assessed with questions of conditioning and everyday physical activity, and physical activity during work trips (hour/week) [18]. The total duration of physical activity was calculated by multiplying the number of sessions of physical activity per week by the duration of each session of physical activity.

#### 2.4.4. Anthropometrics

Height (without shoes), weight (in light indoor clothing) and waist circumference (midpoint between the lowest ribs and iliac bone) were measured by the nurses and body mass index (BMI, kg/m^2^) was calculated.

#### 2.4.5. Biochemical Measurements

Laboratory measurements were taken after a 12 h overnight fast at local healthcare centers and analyzed in their designated quality-controlled laboratories, expect plasma insulin concentration was analyzed in the laboratory of the University of Eastern Finland. The measurements contained fasting, 30 min, and 2 h plasma glucose and insulin concentrations from a 2 h oral glucose tolerance test (OGTT) after the ingestion of 75 g of glucose, blood glycated hemoglobin (HbA1c) as well as fasting plasma total cholesterol, low-density lipoprotein (LDL) cholesterol, high-density lipoprotein (HDL) cholesterol and triglyceride concentrations. Peripheral insulin sensitivity was measured by calculating the Matsuda insulin sensitivity index (MatsudaISI) based on glucose and insulin concentrations at 0, 30 and 120 min of the 2 h OGTT [28,29]. Impaired fasting glucose or impaired glucose tolerance was determined by the American Diabetes Association classification [30].

#### 2.4.6. Other Assessments

Age (years) was calculated based on time of birth and study province and education were based on responses of the StopDia Digital Questionnaire. Education was categorized as “low” (basic education), “medium” (intermediate education) or “high” (higher education).

### 2.5. Statistical Analysis

The study data were managed using REDCap electronic data capture tools hosted at the University of Eastern Finland [31]. Statistical analyses were performed with the SPSS Statistics, version 27.0 (IBM, Armonk, NY, USA). Continuous variables are presented as means (±SD, standard deviation), and categorical variables as frequencies (%). Two-sided *p* values <0.05 were considered statistically significant. We compared baseline characteristics among the groups for continuous normally distributed variables by ANOVA, for non-normally distributed variables by the Kruskal–Wallis *H* test and for categorical variables by the χ2. Cronbach’s alpha coefficients were used to assess internal consistency of the scores of ecSI 2.0^TM^.

Linear mixed-effects models with a maximum likelihood estimation were used to test for the effects of intervention and time on EC. We adjusted the models for sex, age and study province at baseline and included main effects for follow-up time and for study group x follow-up time interaction. These variables were determined as covariates a priori as they may influence EC and other outcome variables. The follow-up time (number of days) was treated as a continuous variable to allow for a small variation in follow-up time between the participants and the different measurements. To study the association of the EC with outcome variables, we entered the total EC score at both time points (baseline, one year) to the model. It was further adjusted for educational level as it may influence EC [9]. Analyses of lipids were also adjusted for medication use for hypercholesterolemia at baseline. To study the independent effect of the change in EC on outcomes, we adjusted the model for the baseline EC score. We also wanted to study independently the effects of the subdomains of EC on outcomes, so they were separately entered in the model without the EC total score. Sub-scores were additionally adjusted for corresponding baseline sub-scores. Regression coefficients, 95% CIs along with *p* values after adjustments and having study group-time interaction in the model, are reported in the tables.

## 3. Results

### 3.1. Characteristics of Particpants

The baseline characteristics of participants are presented in Table 1. The mean (SD) of age was 56 (10) years, and 81% of participants were women. Altogether, 1189 (52%) of participants had obesity (BMI >30 kg/m^2^), and 1241 (54%) had impaired fasting glucose or impaired glucose tolerance. There were no differences in baseline characteristics among the study groups.

### 3.2. Eating Competence at Baseline and Change in Eating Competence over One Year

**Figure 1:**
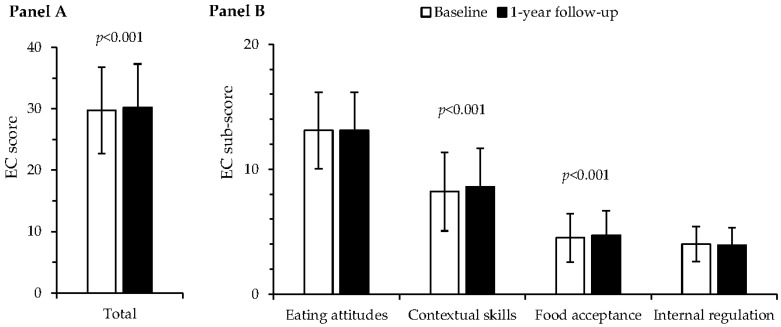
Panel A. Eating Competence (EC) total score (mean ± SD) increased between baseline and one year in the whole study group (*p* < 0.001 for time), but there were no differences between the study groups. Panel B. EC sub-scores (mean ± SD) of contextual skills (*p* < 0.001 for time) and food acceptance (*p* < 0.001 for time) increased between baseline and one year in the whole study group, but there were no differences between the study groups. Linear mixed effects model, *n* = 2291.

The mean (SD) of the EC total score at baseline was 29.7 (7), and there was no difference in the score between the study groups (Table 1). The EC total score increased by 0.5 points (2%) among participants over one year (β = 0.64, 95% CI 0.30–0.97, *p* < 0.001 for time) (Figure 1, Panel A), increasing 0.4 in the digital, 0.5 in the combined digital and group-based and 0.7 in the control group with no difference in the change of the score between the study groups. Altogether 909 (40%) of the participants were defined as eating competent at baseline and 989 (43%) after 1 year with no difference between the study groups.

Of EC sub-scores, contextual skills increased by 0.4 points (5%, β = 0.38, 95% CI 0.22– 0.54, *p* < 0.001 for time) and food acceptance by 0.2 points (4%, β = 0.21, 95% CI 0.11–0.31, *p* = <0.001 for time), whereas eating attitudes and internal regulation did not change (Figure 1, Panel B). There were no differences in the changes of the sub-scores between the study groups.

### 3.3. Change in Eating Competence Total Score Associated with Changes in Diet Quality and Physical Activity

**Figure 2:**
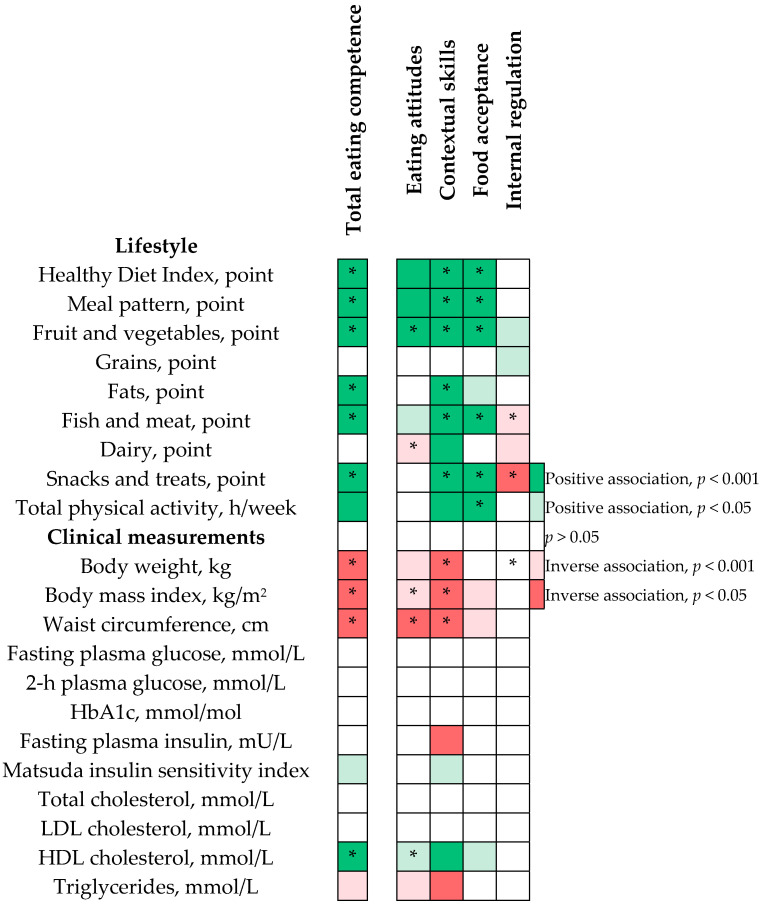
The associations of changes in eating competence (EC) total scores and its sub-scores with Healthy Diet Index, total physical activity and clinical measurements from linear mixed-effects models adjusted for age, sex, study province, educational level, and study group x follow-up time interaction. * The associations were statistically significant after further adjustment for corresponding EC score at baseline. 2-h plasma glucose from oral glucose tolerance test.

An increase in the EC total score was associated with an increase in HDI, its subdomains ‘meal pattern’, ‘fruit and vegetables’, ‘fats’, ‘fish and meat’ and ‘snacks and treats’ as well as total physical activity, all adjusted for age, sex, study province and educational level (Table 2, Figure 2). These associations, except those with the HDI subdomain ‘fats’ and total physical activity, remained statistically significant after further adjustment for the EC total score at baseline (Table 2).

### 3.4. Change in Eating Competence Total Score Associated with Changes in Anthropometry

An increase in the EC total score was associated with a decrease in body weight, BMI, and waist circumference adjusted for age, sex, study province and educational level (Table 2). These associations remained statistically significant after additional adjustment for the EC total score at baseline (Table 2).

### 3.5. Change in Eating Competence Total Score Associated with Changes in Biomarkers of Glucose and Lipid Metabolism

An increase in the EC total score was associated with an increase in MatsudaISI and HDL cholesterol concentration and a decrease in triglyceride concentration adjusted for age, sex, study province and educational level. The association with plasma HDL cholesterol, but not with MatsudaISI or triglycerides, remained statistically significant after further adjustment for the EC total score at baseline (Table 2).

### 3.6. Changes Is Eating Competence Sub-Scores Associated with Changes in Diet and Clinical Characteristics

Increases in EC sub-scores for eating attitudes, contextual skills and food acceptance, but not internal regulation, were associated with an increase in HDI adjusted for age, sex, study province and educational level (Table A1, Figure 2). These associations, except for eating attitudes, remained statistically significant after further adjustment for EC sub-scores at baseline (Table A1). In addition, increases in contextual skills and food acceptance were associated with an increase in total physical activity (Table A1). The association of the increase in contextual skills with total physical activity was no longer statistically significant after additional adjustment for contextual skills at baseline (Table A1).

Increases in eating attitudes, contextual skills and food acceptance were associated with a decrease in BMI and waist circumference after adjustment for age, sex, study province and educational level (Table A1). In addition, increases in contextual skills and eating attitudes were associated with an increase in plasma HDL cholesterol and a decrease in triglycerides concentrations. Moreover, an increase in contextual skills was associated with a decrease in fasting plasma insulin and an increase in MatsudaISI. An increase in eating attitudes was associated with a decrease in waist circumference and an increase in HDL cholesterol, an increase in contextual skills was associated with a decrease in body weight, BMI, and waist circumference and a decrease in internal regulation was associated with an increase in body weight after further adjustment for the corresponding EC sub-scores at baseline (Table A1). A summary of the associations of EC and its sub-scores with outcomes adjusted for age, sex, study province and educational level is presented in Figure 2.

## 4. Discussion

This study is so far the largest to have investigated the effects of a lifestyle intervention on EC and the associations of the changes in EC with diet quality, total physical activity, anthropometrics as well as glucose and lipid metabolism. Our results show that EC slightly improved in adults with an increased risk of T2D who participated in the StopDia study, with no difference between the study groups. Enhanced EC was associated with improved diet quality, decreased body weight, BMI and waist circumference as well as increased plasma HDL cholesterol concentration, regardless of the intervention group or EC at baseline.

Our finding that improved EC was associated with reduced BMI and waist circumference is potentially important for the prevention of obesity and T2D [32,33]. Our observations are also in accordance with the inverse association between EC and BMI shown in many prior cross-sectional studies among different study populations [7,9,10,13,15] as well as with the association between improved EC and weight loss found in a one-year weight management intervention [12]. Moreover, we showed, for the first time, that improved EC was prospectively associated with reduced waist circumference, improved insulin sensitivity as well as increased plasma HDL cholesterol and decreased plasma triglycerides concentrations, which are risk factors of T2D [30]. These results are in line with our previous cross-sectional observations [9]. Even a small weight loss has been shown to be beneficial for the prevention of T2D at population level [34], especially when the usual trend is weight gain [35,36]. If persisting, improved EC could thereby over time help reduce the risk of T2D, even though it was not associated with improved glucose metabolism in our study. Nevertheless, in our previous cross-sectional study with this same study population, better EC was associated with better glucose tolerance [9]. It is thus possible that a longer follow-up time than 1 year or greater change in adiposity would be needed to improve glucose metabolism by improved EC.

To the best of our knowledge, this is the first prospective study to show that improved EC, even regardless of the initial EC level, was associated with improved diet quality, which in turn plays a major role in the prevention of T2D [2,4]. Moreover, improved EC was associated in particular with the increased consumption of fruit and vegetables and improved meal frequency. These prospective results confirm the previous cross-sectional findings from the same study population [9] and, from the other study cohorts [7,8,13]. The observation of the association between improved EC and the increased consumption of fruit and vegetables is important since, for example in Finland, only 14% of working-age men and 22% of women consume the recommended daily amount of fruit and vegetables [37]. Moreover, the consumption of fruit and vegetables as well as dietary patterns characterized by a high consumption of vegetables have been associated with a reduced risk of obesity and T2D [4,38,39]. Our results thus highlight the importance of improvement in EC in lifestyle counselling aimed at preventing T2D.

Our results suggest that, in particular, changes in EC subdomains’ contextual skills, characterized by having regular meals, enough time to eat and considering what kind of foods are good for oneself, as well as food acceptance, characterized by eating a diet with a wide variety of foods, including the new ones, had a beneficial influence on changes in diet quality. Furthermore, the changes in contextual skills and eating attitudes, characterized by a relaxed attitude and enjoyment of eating, had a beneficial influence on the changes in clinical measurements, including those related to weight management. The findings were consistent with our earlier cross-sectional findings [9]. Many of these behaviors and features are also those that have been associated with success in long-term weight management and in the promotion of healthy eating in the previous studies [40,41,42] and should, thus, be emphasized in lifestyle counselling.

The proportion of individuals defined as eating competent increased from 40% to 43% during the study, approaching the higher proportions reported in previous studies among healthy adults, ranging between 39% and 49% [7,8,10,13,15]. Because the increase in EC was rather slight and seen in all study groups, also in the control group, we cannot rule out the possibility that there could have been at least some regression to the mean effect. However, the concomitant changes in EC and various outcome measures including diet quality and cardiometabolic risk factors, support the validity of the finding, although it is challenging to estimate its clinical significance. However, we suggest that if persisting, the clinical significance may increase over the years. Nonetheless, research on long-term changes in EC and its clinical meaning is needed.

It remains uncertain what kind of an intervention should be to enhance EC or its subdomains most efficiently. However, even though the content of any of the intervention groups was not specifically designed to address EC, it included contents in accordance with EC, especially with contextual skills and food acceptance, such as paying attention while eating, trying new foods and eating regularly. These themes are mentioned also in the Finnish nutrition recommendations [27] that was the basis of the written information given to the control group. Thus, as EC was improved in all study groups, including the control group, the different ways to implement or a more intense intervention did not explain the enhancement of EC. Interestingly, however, this suggests that even the mere participation in a lifestyle intervention including increased awareness of the risk of T2D, healthcare visits, receiving laboratory results and information on healthy lifestyle, can be regarded as an intervention. In fact, previous studies have shown that already a brief advice by a physician could contribute weight-related behavior change [43]. Thus, from the healthcare perspective, it is promising that such a minimal intervention could improve EC in adults at increased risk of T2D, at least among those presumably motivated to improve their health behavior.

It should be noted that the associations of improved internal regulation and eating attitudes with a decrease in some components of a healthy diet indicate that the domains of EC were differently associated with diet and health in our study population. However, the ecSI 2.0^TM^ contains only two items for internal regulation, which may affect its reliability. Notwithstanding this, it would be important to investigate whether all aspects of EC are needed to further promote health in adult populations, or whether some subdomains are particularly important. However, according to our results, the influence of improved EC is not limited to diet, but also to total physical activity, which may further mediate the beneficial associations of EC with cardiometabolic health [4,44].

The study has some strengths and limitations. As a strength, it included extensive measurements of glucose metabolism and did not only measure body weight but also waist circumference to assess abdominal adiposity. However, as a limitation it is important to note that the present study does not report the effect of the intervention on weight and glucose metabolism, because they are outcomes of the whole StopDia intervention [18] and will be reported accordingly in the paper including all study participants, also including those without EC data. As another limitation, EC was measured only twice during the study, hence short-term changes in EC could not be evaluated and we cannot rule out the possibility of reverse causality between EC and diet quality. In the future, it would be important to measure EC more frequently during the study and to have a longer follow-up. Due to the nature of the study, diet quality and physical activity were assessed by self-reported questionnaires [18,45], which may not estimate them correctly [45,46,47]. Moreover, diet quality was assessed by HDI [26], which, unlike food diaries, does not provide nutrient-level information. However, our HDI was recently validated against food diaries and was shown to be feasible in healthcare and in large population-based studies [26]. It should be noted also that these results can be generalized only with caution to a general population as all participants were at increased risk of T2D, only a minority of them were men, and they were slightly more educated than Finns on average [48]. On the other hand, sex and the educational level were controlled for in the analyses making them more generalizable. Moreover, a third of Finns aged 50–59 years have a moderate risk of T2D based on the FINDRISC [48], supporting the relevance of the current study population.

## 5. Conclusions

Our results show that participation in a 1-year lifestyle intervention study conducted in healthcare can slightly enhance EC in individuals at risk of T2D, independent of the way of implementation or intensity of the intervention. Moreover, enhanced EC was associated with beneficial changes in risk factors for T2D, including improved diet quality, decreased BMI and waist circumference as well as increased plasma HDL cholesterol. These findings suggest that EC could be a potential target in lifestyle interventions aiming to improve cardiometabolic health and prevent T2D.

## Figures and Tables

**Table 1:** Baseline characteristics of participants.

Descriptive Variable	Control (*n* = 778)	Digital (*n* = 751)	Combined Digital and Group-Based (*n* = 762)
Sex, women	628 (81%)	601 (80%)	617 (81%)
Age, years	56.2 (9.3)	55.8 (9.6)	56.4 (9.6)
Native country, Finland	771 (99%)	744 (99%)	756 (99%)
Study province			
Northern Savo	220 (28%)	200 (27%)	216 (28%)
Southern Carelia	204 (26%)	213 (28%)	210 (28%)
Päijät-Häme	354 (46%)	338 (45%)	336 (44%)
Education level			
Low	53 (7%)	40 (5%)	64 (8%)
Medium	211 (27%)	206 (27%)	192 (25%)
High	514 (66%)	505 (67%)	506 (66%)
Total physical activity, h/week *	10.4 (11.1)	10.2 (9.0)	9.7 (8.6)
Healthy Diet Index (range 0–100) *	63.0 (10.6)	62.8 (11.3)	63.0 (10.9)
Use of cholesterol lowering medication	141 (18%)	129 (17%)	131 (17%)
Body weight, kg	86.4 (16.6)	85.4 (16.9)	85.2 (17.3)
Body mass index, kg/m^2^	31.1 (5.4)	30.7 (5.3)	30.7 (5.4)
Waist circumference, cm	102 (13)	100 (13)	101 (13)
Fasting plasma glucose, mmol/L *	5.60 (0.56)	5.55 (0.56)	5.59 (0.57)
2-h plasma glucose, mmol/L *	6.38 (1.76)	6.26 (1.68)	6.45 (1.73)
EC total score (range 0–48)	29.8 (7.0)	29.8 (6.9)	29.6 (7.2)
Median (IQR)	30 (26–34)	30 (25–35)	30 (25–34)
Eating attitudes (range 0–18)	13.1 (3.0)	13.1 (3.0)	13.1 (3.2)
Median (IQR)	13 (11–15)	13 (11–15)	13 (11–15)
Contextual skills (range 0–15)	8.2 (3.2)	8.1 (3.1)	8.1 (3.1)
Median (IQR)	9 (6–10)	9 (6–10)	8 (6–10)
Food acceptance (range 0–9)	4.4 (1.9)	4.5 (1.9)	4.5 (2.0)
Median (IQR)	4 (3–6)	5 (3–6)	4 (3–6)
Internal regulation (range 0–6)	4.0 (1.4)	4.0 (1.4)	3.9 (1.4)
Median (IQR)	4 (3–5)	4 (3–5)	4 (3–5)
Eating competent (score ≥ 32 from ecSI 2.0^TM^)	307 (40%)	308 (41%)	294 (39%)

Data are frequencies (%) or means (standard deviations, SD). IQR = interquartile range. Eating competence (EC) was measured by the Satter Eating Competence Inventory 2.0^TM^; * Data not available for all participants, *n* = 2252 for physical activity, *n* = 2261 for Healthy Diet Index, *n* = 2290 for waist circumference, *n* = 2284 for fasting plasma glucose, *n* = 2282 for 2 h plasma glucose.

**Table 2:** Associations of changes in eating competence (EC) total score with changes in lifestyle and metabolic factors over one year.

	Model 1		Model 2	
Outcome Measures	β (95% CI)	*p* Value	β (95% CI)	*p* Value
Diet Quality				
Healthy Diet Index, points	0.25 (0.21 to 0.30)	<0.001	0.18 (0.13 to 0.24)	<0.001
Meal pattern	0.03 (0.02 to 0.03)	<0.001	0.02 (0.01 to 0.02)	<0.001
Fruit and vegetables	0.15 (0.12 to 0.17)	<0.001	0.10 (0.07 to 0.13)	<0.001
Grains	0.01 (−0.01 to 0.03)	0.160	0.02 (−0.01 to 0.04)	0.273
Fats	0.03 (0.02 to 0.04)	<0.001	0.02 (−0.001 to 0.04)	0.059
Fish and meat	0.03 (0.02 to 0.04)	<0.001	0.02 (0.01 to 0.03)	0.001
Dairy	−0.002 (−0.01 to 0.01)	0.601	−0.003 (−0.02 to 0.01)	0.660
Snacks and treats	0.02 (0.01 to 0.03)	<0.001	0.02 (0.01 to 0.04)	0.001
Physical activity				
Total physical activity, h/week	0.08 (0.04 to 0.12)	<0.001	0.03 (−0.03 to 0.09)	0.291
Anthropometry				
Body weight, kg	−0.06 (−0.08 to −0.03)	<0.001	−0.04 (−0.07 to −0.01)	0.008
Body mass index, kg/m^2^	−0.02 (−0.03 to −0.01)	<0.001	−0.01 (−0.03 to −0.004)	0.006
Waist circumference, cm	−0.10 (−0.13 to −0.06)	<0.001	−0.06 (−0.10 to −0.02)	0.001
Glucose metabolism				
HbA1c, mmol/mol	−0.01 (−0.02 to 0.01)	0.259	−0.01 (−0.03 to 0.02)	0.616
Fasting plasma glucose, mmol/L	−0.0004 (−0.003 to 0.002)	0.706	0.002 (−0.002 to 0.005)	0.342
2-h plasma glucose from OGTT, mmol/L	−0.002 (−0.01 to 0.01)	0.623	0.01 (−0.01 to 0.02)	0.371
Fasting plasma insulin, mU/L	−0.04 (−0.09 to 0.005)	0.079	0.03 (−0.04 to 0.10)	0.450
Matsuda insulin sensitivity index	0.04 (0.01 to 0.08)	0.011	0.02 (−0.03 to 0.06)	0.404
Lipid metabolism				
Total cholesterol, mmol/L	0.001 (−0.003 to 0.005)	0.614	−0.001 (−0.01 to 0.004)	0.727
LDL cholesterol, mmol/L	−0.0001 (−0.004 to 0.003)	0.972	−0.002 (−0.01 to 0.003)	0.506
HDL cholesterol, mmol/L	0.002 (0.001 to 0.004)	<0.001	0.002 (0.001 to 0.004)	0.005
Triglycerides, mmol/L	−0.004 (−0.01 to −0.001)	0.009	−0.001 (−0.005 to 0.003)	0.629

The data are regression coefficients (β) and their 95% confidence intervals (CI) for changes in outcomes from linear mixed-effects models and *p* values for EC; OGTT = oral glucose tolerance test; Model 1 was adjusted for age, sex, study province, educational level and study group x follow-up time interaction (follow-up time between nurse visits, filling out questionnaires or laboratory visits). Measures of lipid metabolism were also adjusted for cholesterol-lowering medication; Model 2 was additionally adjusted for EC total score at baseline.

## Data Availability

The datasets used and analyzed during the current study are available from the corresponding author on reasonable request. For data sharing inquiries, contact J.P.

## Appendix A

**Table A1:** Associations of changes in eating competence (EC) sub-scores with changes in lifestyle and metabolic factors over one year.

		Eating Attitudes		Contextual Skills		Food Acceptance		Internal Regulation	
		β (95% CI)	*p* Value	β (95% CI)	*p* Value	β (95% CI)	*p* Value	β (95% CI)	*p* Value
Diet quality									
Healthy Diet Index	Model 1	0.18 (0.08 to 0.28)	<0.001	0.76 (0.67 to 0.85)	<0.001	0.72 (0.57 to 0.87)	<0.001	0.05 (−0.14 to 0.24)	0.611
	Model 2	0.12 (−0.0002 to 0.24)	0.050	0.55 (0.43 to 0.66)	<0.001	0.48 (0.30 to 0.67)	<0.001	0.01 (−0.21 to 0.24)	0.902
Meal pattern	Model 1	0.03 (0.02 to 0.04)	<0.001	0.08 (0.07 to 0.09)	<0.001	0.05 (0.04 to 0.07)	<0.001	0.02 (−0.004 to 0.04)	0.114
	Model 2	0.01 (−0.003 to 0.03)	0.122	0.05 (0.03 to 0.06)	<0.001	0.04 (0.01 to 0.06)	0.002	0.01 (−0.02 to 0.04)	0.343
Fruit and vegetables	Model 1	0.14 (0.09 to 0.19)	<0.001	0.38 (0.33 to 0.42)	<0.001	0.42 (0.35 to 0.50)	<0.001	0.13 (0.03 to 0.23)	0.010
	Model 2	0.09 (0.03 to 0.16)	0.006	0.26 (0.19 to 0.32)	<0.001	0.23 (0.13 to 0.33)	<0.001	0.11 (−0.01 to 0.23)	0.078
Grains	Model 1	0.02 (−0.03 to 0.07)	0.392	0.02 (−0.03 to 0.06)	0.448	0.01 (−0.06 to 0.08)	0.757	0.12 (0.03 to 0.22)	0.007
	Model 2	0.03 (−0.03 to 0.09)	0.413	0.01 (−0.04 to 0.07)	0.639	0.03 (−0.06 to 0.12)	0.538	0.08 (−0.03 to 0.19)	0.130
Fats	Model 1	0.01 (−0.02 to 0.04)	0.575	0.10 (0.07 to 0.13)	<0.001	0.05 (0.0002 to 0.10)	0.049	0.04 (−0.02 to 0.11)	0.196
	Model 2	−0.001 (−0.04 to 0.04)	0.948	0.07 (0.03 to 0.11)	0.001	0.03 (−0.03 to 0.10)	0.350	0.04 (−0.04 to 0.12)	0.330
Fish and meat	Model 1	0.03 (0.01 to 0.05)	0.005	0.10 (0.08 to 0.12)	<0.001	0.11 (0.09 to 0.14)	<0.001	−0.06 (−0.10 to −0.02)	0.002
	Model 2	0.02 (−0.005 to 0.04)	0.126	0.07 (0.05 to 0.10)	<0.001	0.04 (0.006 to 0.08)	0.022	−0.07 (−0.11 to −0.02)	0.003
Dairy	Model 1	−0.03 (−0.05 to −0.01)	0.001	0.04 (0.02 to 0.06)	<0.001	0.004 (−0.03 to 0.03)	0.814	−0.07 (−0.11 to −0.03)	0.001
	Model 2	−0.03 (−0.06 to −0.002)	0.037	0.02 (−0.005 to 0.05)	0.103	0.01 (−0.04 to 0.05)	0.736	−0.04 (−0.09 to 0.01)	0.116
Snacks and treats	Model 1	0.003 (−0.02 to 0.03)	0.832	0.10 (0.08 to 0.12)	<0.001	0.09 (0.06 to 0.13)	<0.001	−0.12 (−0.17 to −0.07)	<0.001
	Model 2	0.01 (−0.02 to 0.04)	0.435	0.09 (0.06 to 0.12)	<0.001	0.10 (0.05 to 0.14)	<0.001	−0.10 (−0.16 to −0.05)	<0.001
Physical activity									
Total physical activity, h/week	Model 1	0.04 (−0.06 to 0.14)	0.417	0.24 (0.14 to 0.33)	<0.001	0.36 (0.22 to 0.51)	<0.001	−0.11 (−0.31 to 0.08)	0.257
	Model 2	−0.02 (−0.15 to 0.11)	0.764	0.12 (−0.01 to 0.25)	0.070	0.27 (0.07 to 0.47)	0.009	−0.16 (−0.40 to 0.09)	0.209
Anthropometry									
Body weight, kg	Model 1	−0.08 (−0.14 to −0.02)	0.009	−0.17 (−0.23 to −0.11)	<0.001	−0.09 (−0.18 to 0.004)	0.061	0.10 (−0.01 to 0.21)	0.075
	Model 2	−0.06 (−0.12 to 0.01)	0.072	−0.13 (−0.19 to −0.07)	<0.001	−0.08 (−0.18 to 0.02)	0.113	0.12 (0.002 to 0.23)	0.046
Body mass index, kg/m^2^	Model 1	−0.04 (−0.06 to −0.01)	0.001	−0.07 (−0.09 to −0.04)	<0.001	−0.04 (−0.07 to −0.001)	0.041	0.03 (−0.01 to 0.07)	0.111
	Model 2	−0.02 (−0.05 to <−0.001)	0.050	−0.05 (−0.07 to −0.02)	<0.001	−0.03 (−0.06 to 0.01)	0.121	0.04 (−0.0002 to 0.08)	0.051
Waist circumference, cm	Model 1	−0.18 (−0.26 to −0.10)	<0.001	−0.24 (−0.32 to −0.17)	<0.001	−0.17 (−0.29 to −0.05)	0.005	0.09 (−0.05 to 0.23)	0.226
	Model 2	−0.13 (−0.21 to −0.05)	0.002	−0.15 (−0.23 to −0.07)	<0.001	−0.12 (−0.25 to 0.003)	0.056	0.12 (−0.03 to 0.27)	0.115
Glucose metabolism									
Fasting plasma glucose, mmol/L	Model 1	−0.001 (−0.01 to 0.004)	0.579	−0.002 (−0.01 to 0.003)	0.375	0.003 (−0.01 to 0.01)	0.509	0.002 (−0.01 to 0.01)	0.729
	Model 2	0.002 (−0.005 to 0.01)	0.506	0.002 (−0.005 to 0.01)	0.577	0.004 (−0.01 to 0.01)	0.506	0.003 (−0.01 to 0.02)	0.596
2-h plasma glucose from OGTT, mmol/L	Model 1	−0.005 (−0.02 to 0.01)	0.567	0.003 (−0.01 to 0.02)	0.749	−0.02 (−0.04 to 0.01)	0.278	−0.01 (−0.04 to 0.03)	0.768
	Model 2	0.005 (−0.02 to 0.03)	0.704	0.02 (−0.004 to 0.04)	0.111	−0.02 (−0.05 to 0.02)	0.342	0.02 (−0.03 to 0.06)	0.459
HbA1c, mmol/mol	Model 1	−0.02 (−0.05 to 0.02)	0.385	−0.03 (−0.07 to 0.002)	0.064	0.001 (−0.05 to 0.05)	0.973	0.01 (−0.06 to 0.08)	0.701
	Model 2	−0.02 (−0.07 to 0.02)	0.346	−0.02 (−0.07 to 0.02)	0.314	0.04 (−0.03 to 0.11)	0.240	0.01 (−0.08 to 0.09)	0.903
Fasting plasma insulin, mU/L	Model 1	0.02 (−0.09 to 0.13)	0.729	−0.20 (−0.31 to −0.09)	<0.001	−0.13 (−0.29 to 0.04)	0.127	0.09 (−0.13 to 0.31)	0.402
	Model 2	0.08 (−0.08 to 0.23)	0.333	0.03 (−0.13 to 0.18)	0.737	−0.02 (−0.26 to 0.22)	0.887	0.06 (−0.23 to 0.34)	0.686
Matsuda insulin sensitivity index	Model 1	0.06 (−0.01 to 0.14)	0.115	0.13 (0.05 to 0.20)	0.001	0.06 (−0.06 to 0.17)	0.322	0.02 (−0.13 to 0.17)	0.807
	Model 2	0.05 (−0.05 to 0.14)	0.313	0.03 (−0.07 to 0.12)	0.574	0.02 (−0.13 to 0.16)	0.802	0.04 (−0.14 to 0.21)	0.681
Lipid metabolism									
Total cholesterol, mmol/L	Model 1	−0.0002 (−0.01 to 0.01)	0.960	−0.002 (−0.01 to 0.01)	0.578	0.01 (−0.004 to 0.02)	0.193	0.02 (−0.002 to 0.03)	0.075
	Model 2	−0.003 (−0.01 to 0.01)	0.579	−0.01 (−0.02 to 0.01)	0.293	0.01 (−0.01 to 0.02)	0.430	0.01 (−0.01 to 0.03)	0.388
LDL cholesterol, mmol/L	Model 1	−0.002 (−0.01 to 0.01)	0.585	−0.004 (−0.01 to 0.004)	0.368	0.01 (−0.01 to 0.02)	0.288	0.01 (−0.005 to 0.03)	0.173
	Model 2	−0.01 (−0.02 to 0.004)	0.244	−0.004 (−0.01 to 0.01)	0.417	0.01 (−0.01 to 0.02)	0.503	0.004 (−0.02 to 0.02)	0.712
HDL cholesterol, mmol/L	Model 1	0.005 (0.002 to 0.01)	0.001	0.01 (0.002 to 0.01)	<0.001	0.004 (0.0002 to 0.01)	0.040	0.0004 (−0.005 to 0.01)	0.877
	Model 2	0.005 (0.002 to 0.01)	0.001	0.003 (−0.0005 to 0.01)	0.093	0.004 (−0.001 to 0.01)	0.129	0.001 (−0.005 to 0.01)	0.708
Triglycerides, mmol/L	Model 1	−0.01 (−0.01 to −0.0001)	0.048	−0.01 (−0.02 to −0.01)	<0.001	−0.01 (−0.02 to 0.003)	0.279	0.01 (−0.003 to 0.02)	0.126
	Model 2	−0.004 (−0.01 to 0.004)	0.317	−0.01 (−0.01 to 0.002)	0.163	0.003 (−0.01 to 0.02)	0.663	0.01 (−0.002 to 0.03)	0.095

The data are regression coefficients (β) and their 95% confidence intervals (CI) for changes in EC sub-scores from linear mixed-effects models and p-values for EC sub-scores; Model 1 was adjusted for age, sex, study province, educational level, and study group x follow-up time interaction (follow-up time between nurse visits, filling out questionnaires or laboratory visits). Measures of lipid metabolism were also adjusted for cholesterol-lowering medication; Model 2 was additionally adjusted for corresponding EC sub-scores at baseline.
